# Emerging applications of read profiles towards the functional annotation of the genome

**DOI:** 10.3389/fgene.2015.00188

**Published:** 2015-05-19

**Authors:** Sachin Pundhir, Panayiota Poirazi, Jan Gorodkin

**Affiliations:** ^1^Center for non-coding RNA in Technology and Health, Department of Veterinary Clinical and Animal Sciences (IKVH), University of CopenhagenFrederiksberg C, Denmark; ^2^Computational Biology Lab, Institute of Molecular Biology and Biotechnology, Foundation for Research and Technology-HellasHeraklion, Greece

**Keywords:** read profile, RNA-seq, non-coding RNA, ChIP-seq, enhancer, patterns

## Abstract

Functional annotation of the genome is important to understand the phenotypic complexity of various species. The road toward functional annotation involves several challenges ranging from experiments on individual molecules to large-scale analysis of high-throughput sequencing (HTS) data. HTS data is typically a result of the protocol designed to address specific research questions. The sequencing results in reads, which when mapped to a reference genome often leads to the formation of distinct patterns (read profiles). Interpretation of these read profiles is essential for their analysis in relation to the research question addressed. Several strategies have been employed at varying levels of abstraction ranging from a somewhat *ad hoc* to a more systematic analysis of read profiles. These include methods which can compare read profiles, e.g., from direct (non-sequence based) alignments to classification of patterns into functional groups. In this review, we highlight the emerging applications of read profiles for the annotation of non-coding RNA and *cis*-regulatory elements (CREs) such as enhancers and promoters. We also discuss the biological rationale behind their formation.

## Introduction

Advances in high-throughput sequencing (HTS) technologies have revolutionized the field of molecular biology. Two widely used experimental protocols derived from this technology are: (a) **RNA sequencing (RNA-seq)**; and, (b) **Chromatin immunoprecipitation coupled with DNA sequencing (ChIP-seq)** reflecting proteins interacting with DNA. Both of these protocols are designed to sequence a biological molecule, which in case of RNA-seq is RNA and in case of ChIP-seq is DNA, extracted from a sample of interest (Johnson et al., 2007; Morin et al., 2008). More specifically, RNA-seq allows the capture and determination of the nucleotide sequence of different RNA molecules, which can be short or long RNA, RNA having 3′ poly-A tail (typically messenger RNA) or total RNA (complete transcriptome, typically excluding ribosomal RNA) (Morin et al., 2008). In contrast, ChIP-seq experiments facilitate the capture and determination of the nucleotide sequence of specific DNA fragments, which typically are part of genomic regions where a specific protein interacts with the DNA (Johnson et al., 2007). ChIP-seq is typically used to determine how transcription factors influence phenotype-affecting mechanisms (Johnson et al., 2007).

KEY CONCEPT 1. RNA Sequencing (RNA-seq) Method based on high-throughput sequencing technology that is used to determine the nucleotide sequence of all the RNAs transcribed within a given sample (typically, cell line or tissue).

KEY CONCEPT 2. Chromatin immunoprecipitation coupled with DNA sequencing (ChIP-seq) Method based on high-throughput sequencing technology that is used to determine the nucleotide sequence of all the DNA segments in the genome where a protein interacts.

**Figure 1:**
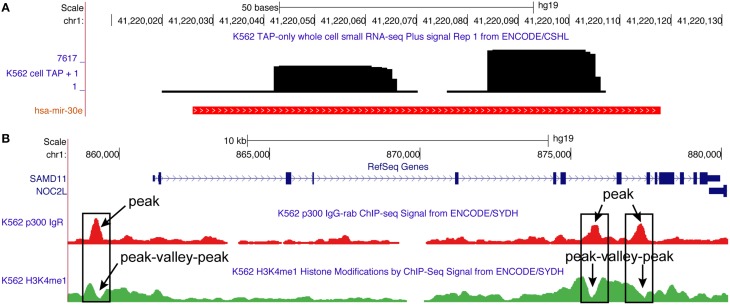
**Typical read profiles obtained for microRNA (has-mir-30e) and transcription factor (P300) from the small RNA-seq and ChIP-seq data, respectively. (A)** Read profile for hsa-mir-30e is observed in K562 cell line using small RNA-seq data from the ENCODE project (Fejes-Toth et al., 2009). It consists of two read blocks corresponding to two arms (passenger and mature) of the microRNA. **(B)** Read profile for P300 transcription factor is observed in K562 cell line using ChIP-seq data from the ENCODE project (Euskirchen et al., 2007). It consists of a peak that signify the position where P300 binds to the genome. Also shown is the peak-valley-peak read profile for histone modification (H3K4me1) observed at P300 peaks (O'Geen et al., 2011). Both P300 (peak) and H3K4me1 (peak-valley-peak) read profiles are enriched at enhancer regions (Merika et al., 1998; Heintzman et al., 2007), and are thus useful for their annotation in the genome.

A common end product of both these protocols is millions of nucleotide sequences, generally referred to as “**reads**.” These reads carry the nucleotide sequence information of various RNA and DNA molecules captured during the RNA-seq and ChIP-seq experiments, respectively. To determine the genomic location of these reads, they are mapped back to the reference genome using mapping tools (see e.g., Fonseca et al., 2012; Otto et al., 2014). During mapping, a read is assigned to its genomic location based on the similarity between the nucleotide sequence of reads and the genomic region, respectively. Once mapped, a coverage pattern of the number of reads mapping at each nucleotide position of the reference genome is obtained. The coverage pattern for a specific genomic region (locus) or a transcript is referred to as a “**read profile**” (Langenberger et al., 2012). Thus a read profile, essentially, represents the positional arrangements of reads onto a specific region in the genome (Figure 1). Recently, a number of computational methods have been developed that utilize the concept of read profiles for functional analysis. We also previously reported the application of read profiles, obtained from short RNA-seq data, for the efficient prediction of microRNAs (miRNAs) (Pundhir and Gorodkin, 2013). Here, we discuss the wider application of read profiles by extending it from the annotation of miRNAs to other non-coding RNAs (ncRNAs) as well as *cis*-regulatory elements (CREs), such as enhancers and promoters. Specifically, we review how different computational methods exploit read profiles obtained from RNA-seq and ChIP-seq data for the functional annotation of ncRNA and CREs, respectively (Table 1). We also discuss the biological rationale behind the generation of various read profiles.

KEY CONCEPT 3. Read Nucleotide sequence of a RNA or DNA determined using RNA-seq or ChIP-seq, respectively.

KEY CONCEPT 4. Read profile Coverage pattern showing the number of reads mapping at each nucleotide position of a distinct region in the reference genome.

**Table 1:** **A brief summary of computational methods that use the concept of read profiles for the prediction of microRNA (miRNA), non-coding RNA (ncRNA) and *cis*-regulatory elements (CRE)**.

**Application^a^**	**Method^b^**	**Data source^c^**	**Read profile characteristic^d^**	**Methodology^e^**
Micro-RNA prediction	miRDeep, miRDeep2, miRDeep^*^	Short RNA-seq	Two predominant cluster of reads corresponding to mature and passenger miRNA strand	Bayesian statistics, along with stable hairpin loop secondary structure (Friedländer et al., 2008, 2012; An et al., 2013)
	miRanalyzer			Random forest classifier, along with stable hairpin loop secondary structure (Hackenberg et al., 2009)
	miRdba			Optimal alignment of candidate and known miRNA read profiles (Pundhir and Gorodkin, 2013)
Non-coding RNA classification	Langenberger et al.	Short RNA-seq	Varying number of read clusters separated by specific number of nucleotides for major ncRNA classes (miRNA, snoRNA and tRNA). The reads are often arranged at different degree of precision (entropy)	Random forest classifier trained on different read profile features (length, expression and others) to classify miRNA, snoRNA and tRNA (Langenberger et al., 2010)
	Jung et al.			Length and expression depth of read profiles, followed by motif and sequence similarity analysis to predict snRNA and snoRNA (Jung et al., 2010)
	deepBlockAlign, ALPS			Optimal alignment between two read profiles to classify miRNA, snoRNA and tRNA (Erhard and Zimmer, 2010; Langenberger et al., 2012)
	BlockClust			Graph-kernel trained on different read profile features such as minimum read length and entropy to classify miRNA, snoRNA and tRNA (Videm et al., 2014)
*cis*-regulatory element prediction	DFilter	TF ChIP-seq	Reads arranged in the form of a peak profile	Hotelling observer based on signal processing to detect regions enriched for peaks (Kumar et al., 2013)
	Kaikkonen et al.	Histone ChIP-seq	Reads arranged in the form of a peak-valley-peak read profile	Sliding window approach to detect peak-valley-peak read profile in order to measure spatiotemporal activity of CRE (Kaikkonen et al., 2013)
	CAGT			Pearson correlation coefficient between read profiles that are represented in the form of vector of signal values. Read profiles having high correlation are clustered together (Kundaje et al., 2012)
Detect novel ncRNA classes or known ncRNAs (potentially different) sharing similar processing	deepBlockAlign, ALPS	Short RNA-seq	Read profile characteristics (such as number of read clusters and length) shared by two or more transcripts	Optimal alignment between two read profiles (Erhard and Zimmer, 2010; Langenberger et al., 2012)

*Also included are two methods that can detect novel ncRNA classes or known ncRNAs sharing similar processing based on the similarity in their corresponding read profiles*.

a *The application of the computational method*.

b *Name or the literature reference of the computational method*.

c *High-throughput sequencing data that is used by the method for analysis*.

d *Characteristic of read profiles that the method exploits*.

e *Brief description of the computational technique used behind the method*.

## Toward functional annotation of small non-coding RNA using read profiles

A substantial fraction of the HTS data is used for the analysis of **non-coding RNAs (ncRNAs)**. This is in part due to the large potential for ncRNAs, which was first realized with the sequencing of the human genome which revealed that only ~1.2% of genome encodes for proteins (Lander et al., 2001). In complement the recent ENCODE project, based on RNA-seq experiments suggests that ~75% of the human genome is transcribed into RNA (Djebali et al., 2012). Still there is an ongoing debate on the degree at which the abundance of transcripts should be measured, since for e.g., long non-coding RNA (lncRNA) are expressed at much lower levels compared to mRNAs (Eddy, 2014). Obviously the large fraction of noncoding transcripts does not directly imply that they are all functional. In fact, the exact fraction of ncRNA that is actually functional is thus far not understood and is subject to much debate within the scientific community (Doolittle, 2013; Graur et al., 2013). The recent GENCODE effort (v22) identified 15,900 lncRNAs and ~10,000 small ncRNA genes (http://www.gencodegenes.org/stats/current.html) (Derrien et al., 2012) from careful transcript analysis. However, the vast majority of the lncRNAs have not yet been assigned a function (Mattick and Rinn, 2015).

KEY CONCEPT 5. Non-coding RNAs (ncRNAs) RNA molecules transcribed from their respective genes, but not translated into proteins.

An important step toward uncovering the function of non-coding transcripts includes the study of their read profiles. The read profiles can be linked with RNA secondary structure, in particular for miRNAs and sometimes also for tRNAs and snoRNAs (Kawaji et al., 2008; Langenberger et al., 2010).

**Figure 2:**
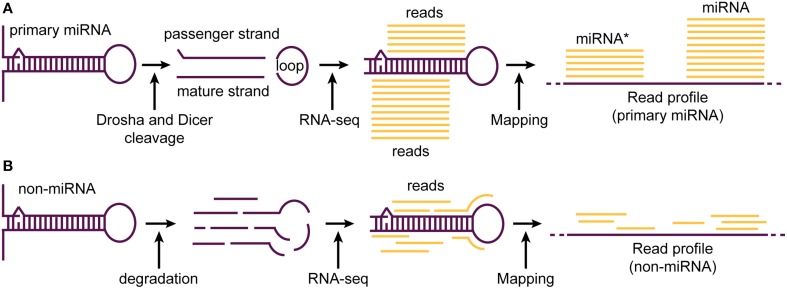
**A typical read profile generated upon the processing of miRNA and random processing (degradation) of a non-miRNA transcript. (A)** Primary miRNA transcript is precisely processed by Drosha and Dicer enzymes leading to the generation of a ~22 nt duplex (passenger and mature strand) and a loop region. While, the mature miRNA is protected from degradation by being incorporated into the miRNA-induced silencing complex (miRISC), both passenger and loop region are mostly degraded. Therefore, most reads obtained during short RNA-seq experiments correspond to the mature miRNA strand. When mapped to the reference genome, reads corresponding to miRNA and star miRNA align in a pattern (read profile) consisting of two major read clusters sharing almost the same 5′ end base position. **(B)** In contrast to precise processing of miRNA transcript, non-miRNA transcripts are processed at random base positions. This leads to the generation of many RNA fragments of no fixed length. When sequenced during RNA-seq experiments and mapped to the reference genome, the generated read profile consists of randomly placed reads having high variability in their 5′ end base positions.

MiRNAs form probably one of most studied class of non-coding RNA due to its widely recognized role in regulating the expression of genes (Bartel, 2009). It is estimated that 30–60% of all the human protein coding transcripts are targeted by one or more miRNAs in one or more cellular contexts (Krek et al., 2005; Friedman et al., 2009). MiRNAs are small ncRNA (18–24 nucleotides) that are crucial in various biological and metabolic pathways. The majority of animal miRNAs are transcribed as long primary transcripts from which one or more ~70 nt long hairpin precursors (pre-miRNAs) are cleaved out by the Drosha endonuclease (Winter et al., 2009). The pre-miRNAs are exported to the cytosol where they are cleaved by the Dicer protein, releasing the loop of the hairpin and a ~22 nt duplex consisting of the mature miRNA and the star miRNA (Figures 1A, 2). The duplex is unwound and the mature miRNA is incorporated into the miRNA-induced silencing complex (miRISC), which can transfer it to target sites in the 3′ UTRs of mRNA transcripts. This effector complex then regulates the expression of target genes by directly cleaving targeted mRNAs (Kawasaki and Taira, 2004) or repressing their translation (Williams, 2008).

### Read profiles to annotate MicroRNAs

Most of the initial efforts for computational prediction of miRNA utilized characteristic hairpin secondary structure of miRNA with homology search (Wang et al., 2005; Dezulian et al., 2006) or evolutionary conservation (Lai et al., 2003; Lim et al., 2003). Also methods based on phylogenetic shadowing (Berezikov et al., 2011), neighbor step loop search (Ohler et al., 2004), minimal folding free energy index (Zhang et al., 2006), machine learning (Oulas et al., 2009; Karathanasis et al., 2015), and statistical approaches (Gkirtzou et al., 2010; Karathanasis et al., 2014) have been developed. A major drawback of these methods is that they require that the novel miRNAs should either share similar sequence (homology based method) or certain characteristic features (for statistical and machine learning methods) with already known miRNAs. The problem is further compounded by recent findings that many miRNA-sized small RNAs can also be produced from other classes of structured RNAs like snoRNA and tRNA (Ender et al., 2008; Kawaji et al., 2008; Cole et al., 2009; Lee et al., 2009; Taft et al., 2009; Haussecker et al., 2010; Brameier et al., 2011; Li et al., 2012b).

High-throughput short RNA-seq experiments that are designed to sequence short RNA fragments (typically <50 nt) have proved ideal to identify novel miRNAs and also to robustly quantify their expression across different physiological conditions (Figures 1A, 2). Due to the large number of reads obtained after a typical short RNA-seq experiment, significant efforts have been made to develop a range of computational methods for their analysis and efficient prediction of miRNAs. A few widely used methods among these efforts are miRDeep (Friedländer et al., 2008), miRDeep2 (Friedländer et al., 2012), miRDeep^*^ (An et al., 2013) and miRanalyzer (Hackenberg et al., 2009). All these methods predict miRNAs based on the charactertistic patterns by which the short reads map to the genome, combined with their secondary structure potential.

The miRDeep and miRDeep2 methods use bayesian statistics to score the fit of sequenced RNAs (reads) to the biological model of miRNA biogenesis. Specifically, they start by mapping reads to known precursor miRNAs and assigning them to corresponding miRNA annotations. Next, they analyse the genomic regions where remaining reads align for their potential as precursor miRNA. This analysis includes: (a) assigning a read with highest expression at a potential miRNA locus as the predicted mature miRNA. This is followed by extending the read by 20 bp (offset miRNA) at one end and by 70 bp (loop, miRNA^*^, offset miRNA) toward the other end; and, (b) identifying a viable hairpin secondary structure corresponding to the defined potential miRNA locus using an RNA secondary stucture prediction method, in this case RNAfold (Lorenz et al., 2011). A log-odds probability score signifying the probability of a precursor sequence to be a genuine miRNA precursor vs. the probability of it being a background hairpin is computed based on bayesian statistics (Friedländer et al., 2008).

Another method, miRanalyzer follows the analysis steps similar to miRDeep for predicting known miRNAs. However, for predicting novel miRNAs, it utilizes several features associated with mapping and secondary structure such as read count, mfe (mean free energy), stem length and loop length to train a random-forest classifier (Hackenberg et al., 2009).

While based on miRDeep, miRDeep^*^ utilizes a different strategy to identify precursor miRNAs. Specifically, it considers the highest expressed read at a potential miRNA locus as the predicted mature miRNA, followed by an extension of 22 bp (offset miRNA) toward one side and subsequent extensions of 15 bp (loop region) and read length (miRNA^*^) and 22 bp (offset miRNA) at the other end (An et al., 2013). This strategy is similar to that adopted in the second version of miRDeep i.e., miRDeep2 (Friedländer et al., 2012).

Overall, from the benchmark reported (98.6% accuracy for miRDeep2, 97.9% accuracy for miRanalyzer) and subsquent discovery of novel miRNAs, e.g., in (Friedländer et al., 2008) all these methods perform well in predicting novel miRNAs. However, they have two major shortcomings: (a) By defining strict length criteria, such as 15 bp for loop region or 22 bp for offset miRNA, these methods tend to miss unconventional miRNAs, like miRNA-offset RNA (moRs) that encode for up to four distinct stable small RNAs (Shi et al., 2009) or novel miRNAs that may not follow this criteria; and, (b) They require a candidate region to fold into a stable haipin secondary structure. Since, RNA secondary stucture prediction methods are not always accurate, especially in regions of low sequence conservation, a genomic region devoid of secondary structure information will be missed as a novel miRNA. Indeed, many mRNA regions that were predicted to form large, single stranded loops by secondary structure prediction method (RNAfold) have been shown to form base-paired regions using experimental methods (Zheng et al., 2010; Li et al., 2012a).

The recently developed method, miRdba address these shortcomings by predicting miRNAs purely based on the pattern by which the short reads map to a certain genomic region (read profile; Figures 1A, 2) (Pundhir and Gorodkin, 2013). Specifically, it utilizes a “**read profile based alignment**” algorithm, deepBlockAlign (Langenberger et al., 2012) to compare read profiles from a candidate region with a database of known miRNA read profiles, compiled using miRBase (Kozomara and Griffiths-Jones, 2011). A candidate region is predicted as a novel miRNA, if the alignment score between the candidate read profile and database is above a benchmarked threshold. On benchmarking, miRdba performed similar to the previously developed methods, miRanalyzer and miRDeep (Pundhir and Gorodkin, 2013). However, miRdba has following advantages: (a) Due to being not dependent on the RNA secondary structure (hapirpin) information, it can predict miRNAs in regions that are devoid of this information. Indeed, miRdba predicted ~500 novel miRNA candidates, most of which were located in low evolutionary conserved regions of the human genome (Pundhir and Gorodkin, 2013), and; (b) The scores based on the alignment of read profiles can be used to identify distinct clusters of short RNAs sharing similar processing patterns as shown for miRNAs from animals and plants (Pundhir and Gorodkin, 2013) or to identify RNAs from different ncRNA classes sharing similar processing patterns as shown for miRNAs, snoRNAs and tRNAs (Langenberger et al., 2012). Interestingly, the primary online repository of miRNAs, miRBase, has also recently interegrated the concept of read profiles to validate the miRNA entries in the database (Kozomara and Griffiths-Jones, 2011). A primary feature used toward this validation is the presence of consistent 5′ end position of the reads mapping to a given mature miRNA annotation, which can readily be comprehended from a read profile.

KEY CONCEPT 6. Read profile based alignment Optimal alignment of two read profiles such that the mean difference between normalized read counts at their aligned positions is minimum.

### Read profile analysis of small RNA-seq data

The application of read profiles has also been extended to compare processing patterns between two RNAs. Methods like ALPS (Erhard and Zimmer, 2010) and deepBlockAlign (Langenberger et al., 2012) have been developed to compare read profiles. Whereas, one application of these “read profile based alignment” methods is to identify ncRNAs from the same family, another is to search similar local profiles between ncRNAs from different families, with the goal of identifying similar processing as has been observed between for example tRNAs and miRNAs (Cole et al., 2009; Langenberger et al., 2012).

A common motivation is that the read profile is a distinct feature that reflects the processing mechanism of these ncRNA classes and it often depends on their secondary structure. However, different approaches have been used to capture the distinguishing features of read profiles to classify ncRNAs into respecitve families. More specifically, Langenberger et al. (2010) used a random-forest classifier trained on different read profile features (length, expression and others) to classify miRNA, snoRNA and tRNA. The method achieved a recall rate of ~80% for the three ncRNA classes, however the performance was better for miRNA in comparison to tRNA and snoRNA (Langenberger et al., 2010). Another method used only the length and expression depth of read profiles, followed by motif and sequence similarity analysis to predict novel snoRNAs and snRNAs (Jung et al., 2010). Eight out of the 10 novel snoRNA predicted by this method were later confirmed using the Northern blot analysis, showing the strong predictive power of this approach (Jung et al., 2010). The “read profile based alignment” algorithms, ALPS (Erhard and Zimmer, 2010) and deepBlockAlign (Langenberger et al., 2012) were also applied to classify ncRNAs into miRNA, snoRNA and tRNA classes. Both methods showed good perfomance in ncRNA classification. Specifically, ALPS reported a recall and precison of ~90% and ~60%, respectively for both miRNAs and tRNAs. Similarly, deepBlockAlign classified miRNAs and tRNAs into two distinct clusters emanating from well seperated branches of a hierarchical tree (see Figure 4 from Langenberger et al., 2012). Also a sub-class of miRNA, miRNA-offset RNAs (moRs) was located as a distinct sub-cluster within the miRNA cluster at a *p*-value of 0.06.

Besides ncRNA classification, both ALPS and deepBlockAlign also identified many unannotated RNAs, snoRNAs and tRNAs having read profiles similar to that from known miRNAs (Erhard and Zimmer, 2010; Langenberger et al., 2012). This highlights the wider application of these methods to detect RNAs that potentially share similar post-transcriptional processing patterns. Indeed, recent studies based on wet-lab experiments have confirmed that some tRNA and snoRNA can be processed to produce miRNA-sized small RNA fragments (Haussecker et al., 2010; Brameier et al., 2011). A recently published method, BlockClust (Videm et al., 2014), also aims to classify ncRNA into miRNA, snoRNA and tRNA, however unlike ALPS and deepBlockAlign, it is based on a graph-kernel method trained on different read profile features such as minimum read length and entropy. Due to the nature of its supervised training, the prediction of BlockClust is limited to known ncRNA classes, whose read profiles have been used for training the computational model. Furthermore, primarily due to low number of snoRNAs in the input dataset, all the methods discussed above have relatively moderate accuracy in predicting snoRNAs as compared to that reported for miRNAs and tRNAs (Erhard and Zimmer, 2010; Langenberger et al., 2010, 2012; Videm et al., 2014).

## Toward functional annotation of *Cis*-regulatory elements using read profiles obtained from ChiP-seq data

The ***cis*-regulatory elements (CREs)** are distinct positions in the genome that are actively bound by various transcription factors resulting in an increase or decrease in the expression of, mostly, proximal located genes (Wittkopp and Kalay, 2012). Thus, they are involved in tissue-specific expression of genes and include enhancers, promoters, silencers and others. Two important characteristics of CREs are: (a) the presence of one or more nucleotide sequence motifs that define specificity and binding affinity of various transcription factors; and, (b) the marked absence of nucleosome, which is the basic structural unit in which DNA is packed around a histone protein core (Hardison and Taylor, 2012; Mathelier et al., 2015). The first characteristic has been widely used by computational methods for the prediction of CRE (Van Loo and Marynen, 2009). However, these methods have two main disadvantages: first the presence of a sequence motif does not necessarily imply that a region is involved in *cis*-regulation. Due to the low sequence complexity and short length of many of these motifs, they can be observed at thousands of places in the genome based on random permutation, thus leading to many false positive predictions. The second disadvantage is that, even if a sequence motif actually corresponds to a CRE, this does not convey information about the activity level of the CRE in a particular cell type (Elnitski et al., 2006).

KEY CONCEPT 7. *cis*-regulatory elements (CREs) Distinct positions in the genome actively bound by various transcription factors resulting in an increase or decrease in the expression of, mostly, proximal located genes. These include enhancers, promoters, silencers and others.

**Figure 3:**
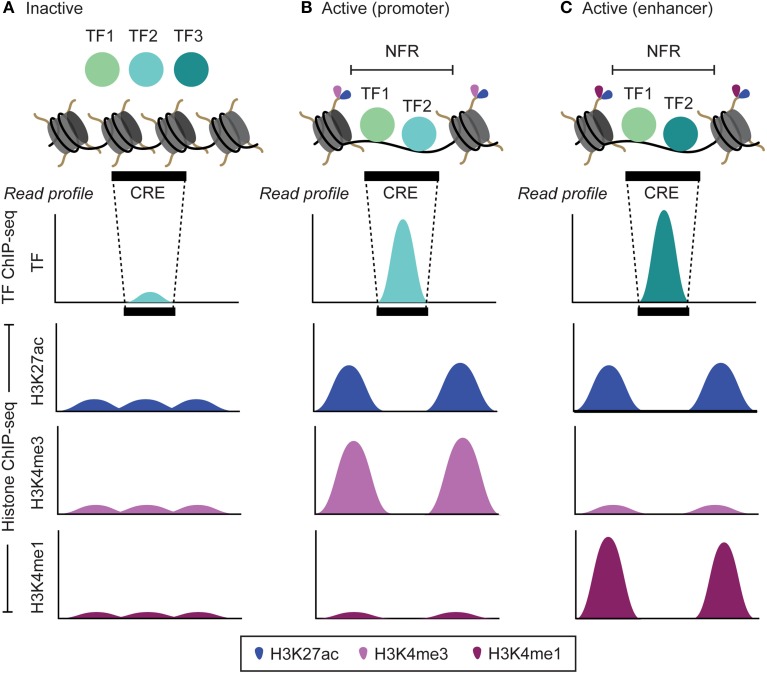
**The transcription factor and histone modification landscape for inactive and active** ***cis*****-regulatory elements (CRE; promoters and enhancers) and the corresponding read profiles. (A)** When inactive, the DNA corresponding to the CRE is wrapped around histone proteins in the form of a basic structural unit termed as nucleosome. This prevents any interaction of transcription factors (TF) with the DNA. **(B,C)** When active, a series of histone modifications (H3K4me1, H3K4me3, H3K27ac and others) along side interaction with specific TF (pioneer factor) make the overlapping nucleosomes at a CRE hypermobile. These nucleosomes are then displaced apart leading to formation of nucleosome free regions (NFRs) that are subsequently bound by TFs. During TF ChIP-seq and Histone ChIP-seq experiment, reads corresponding to TF bound NFRs and histone-modified regions flanking the NFRs are obtained, respectively. Upon mapping, it leads to a read profile in the form of a peak shape for NFRs (TF ChIP-seq) and peak-valley-peak shape for regions flanking the NFRs (Histone ChIP-seq). By analyzing the read intensity in these read profiles, we can determine active CRE, TF bound at these CRE and also the level of their activity. Since, distinct sets of histone modifications are observed at enhancers (H3K27ac and H3K4me1) and promoters (H3K4me3 and H3K4me1), analyzing peak-valley-peak histone read profile also facilitates to distinguish between enhancers and promoters.

The recently developed ChIP-seq technology allows us to address both these shortcomings by exploiting the second characteristic of CRE, which is the marked absence of nucleosomes in these regions (Mathelier et al., 2015) (Figures 1B, 3). When inactive, the genomic region corresponding to a CRE is packed into nucleosomes. Prior to activation, a specific class of transcription factors (pioneer factors) along with coactivator proteins interacts with the nucleosomes to modify their histone composition, such as H3K4me1, H3K4me3, H3K27ac that makes them hypermobile (Zaret and Carroll, 2011) (Figure 3). These histone modifications reflect many aspects of proximal gene expression; for example, trimethylation of histone H3 on lysine 4 (H3K4me3) reflect promoter activity and is highly correlated with the gene expression levels (Figure 3B). Similarly, monomethylation of histone H3 on lysine 4 (H3K4me1) and acetylation of histone H3 on lysine 27 (H3K27ac), are associated with the activity of enhancers (Figure 3C) (Heintzman et al., 2007). During activation, the hypermobile nucleosomes at CRE are displaced apart, thus making the CRE accessible for the assembly of other transcription factors to form a larger protein complex, such as promoter initiation complex (PIC) assembled at the promoters to initiate gene transcription (Shlyueva et al., 2014). A genomic region corresponding to CRE that is devoid of any nucleosomes is referred to as a nucleosome free region (NFR), and is flanked by hypermobile nucleosomes modified for specific histones depending on the class of CRE itself, such as H3K4me1 and H3K4me3 in case of enhancers and promoters, respectively (Figure 3) (Calo and Wysocka, 2013; Shlyueva et al., 2014).

### Read profiles to annotate and measure the activity level of *Cis*-regulatory elements

A typical ChIP-seq experiment is designed to capture and sequence DNA fragments corresponding to: (a) the NFRs bound by a specific transcription factor (TF ChIP-seq), or (b) the region flanking NFRs where the nucleosome undergoes specific histone modifications (Histone ChIP-seq) (Johnson et al., 2007; O'Geen et al., 2011). Upon mapping, the positional arrangement of reads from TF ChIP-seq, typically, leads to a pattern (read profile) characterized by a peak corresponding to the NFRs (Figures 1B, 3). Similarly, the reads from Histone ChIP-seq, typically, lead to a peak-valley-peak pattern (read profile) around the NFRs (Kumar et al., 2013). Correct interpretation of these peak arrangements is crucial for meaningful identification of NFRs or CRE. A common goal after mapping reads from TF ChIP-seq is to be able to distinguish between genuine and spurious peaks in order to robustly identify the genome wide positions where a specific transcription factor is bound *in vivo*. These positions in turn will also reflect the site of active CREs. The recently developed DFilter method detects the enrichment of peaks based on their shapes (read profile) (Kumar et al., 2013). Specifically, it captures the shape using a technique adapted from signal processing, known as Hotelling observer. This technique uses the mean and covariance of mapped read profiles to maximize the difference between filter outputs at true-positive regions and noise regions. On benchmarking using ChIP-seq data from three different cell lines, the method consistently performed better compared to the widely used peak-finding algorithms, such as MACS (Zhang et al., 2008), F-seq (Boyle et al., 2008), and SICER (Zang et al., 2009). Furthermore, unlike MACS and similar methods that are specifically designed for peak finding, the DFilter method performed equally well on other HTS technology data such as DNase-seq and FAIRE-seq to detect NFRs (Kumar et al., 2013). This suggest that methods based on the concept of read profiles can be both robust as well as general for the analysis of a wide range of HTS data. Indeed another recent study showed high performance in predicting CRE (enhancers) using read profiles generated from CAGE data across a wide range of human tissues and cell types (Andersson et al., 2014).

The peak-valley-peak read profile, typically, observed using histone ChIP-seq data has also been used to study the spatiotemporal activity of NFRs across different cell types (Figures 1B, 3). Kaikkonen et al. (2013) studied the epigenetic landscape of NFRs (enhancers) during different time points of macrophage activation. To identify likely NFRs, histone enriched regions in the genome were scanned by comparing the histone read density within 100 bp intervals (valley) relative to the flanking 150 bp regions (peaks). The location with the greatest disparity in read density was assigned as a NFR. Based on this search criterion, authors were able to locate several pre-existing as well as novel enhancers, which are formed only during activation of a specific signaling pathway (Kaikkonen et al., 2013). The peak-valley-peak read profile also enabled visualizing the intermediate stages of NFR formation during different time points of macrophage activation. A similar criterion for NFR analysis has also been used in several recent studies (Heinz et al., 2010, 2013; Pham et al., 2012; Zhang et al., 2012; Kaikkonen et al., 2013; Lara-Astiaso et al., 2014). Taking a step further, Kundaje et al. (2012) unraveled that not only the peak-valley-peak read profile, but also an asymmetry in this profile convey information about the activity of the corresponding NFR. The authors developed a method, CAGT, to study the nucleosome positioning signals around bound transcription factors at transcription start sites (TSS). It not only accounts for the magnitude of the signal but also the shape and implicit strand orientation of histone modification marks (Kundaje et al., 2012). Using the method on 12 histone modifications around the binding sites of 119 transcription factors and nucleosome positioning data around TSS from a large number of cell lines, they unveiled correlation between chromatin marks, nucleosome positioning, and sequence content. More specifically, peak-valley-peak profiles having more pronounced peaks upstream to TSS as compared to downstream regions were associated with higher gene expression. In contrast, the genes having peak-valley-peak profiles at their TSS skewed toward the downstream region showed lower expression. Similarly asymmetry in peak-valley-peak profiles was also observed at the binding sites of 119 transcription factors located distally from the TSS. Many of these sites are enhancers and asymmetry in the read profiles may be of structural importance for the interaction of these sites with other functional elements such as promoters (Kundaje et al., 2012).

## Discussion

Advances in HTS technology have opened several new avenues for the functional annotation of the genome using novel approaches. We have discussed about one such approach that is based on the pattern by which reads map to the reference genome (read profile) for the functional annotation of ncRNAs and CREs (Table 1). Various computational methods have used the concept of read profiles at varying levels of abstraction. Some methods used the read profile features such as expression, length, and distance between consecutive read blocks along with secondary structure information for ncRNA prediction. Others explicitly used the shape represented in a read profile for ncRNA prediction. Similarly, methods inspired from signal processing to shifting window-based approach have been utilized to robustly characterize the read profiles associated with different CREs.

Similar to the interpretation of read profiles, several different methodologies have been used to generate them. As a primary step, reads are aligned to the genome using different alignment tools, such as bowtie as in the case of miRanalyzer, miRDeep2 and miRDeep^*^ (Hackenberg et al., 2009; Friedländer et al., 2012; An et al., 2013). Many of these methods (miRDeep2, ALPS and deepBlockAlign) support other alignment tools, such as BWA (Li and Durbin, 2010), and report similar conclusions (Erhard and Zimmer, 2010; Friedländer et al., 2012; Langenberger et al., 2012). However, a detailed study focusing on the effect of different alignment tools on read profiles has not been performed. Another important parameter is whether to include the reads mapping at multiple positions during the analysis or not. Here, miRDeep2 sets this parameter to upmost five positions (Friedländer et al., 2012) and miRdba analyze only uniquely mapped reads (Pundhir and Gorodkin, 2013). Considering that miRdba only depends on similarity between read profiles for the predictions, it is important to utilize only uniquely mapped reads in order to limit false positive predictions. Similarly, for CRE predictions, collapsing reads mapping at identical positions is recommended in order to limit PCR duplicates (Zhang et al., 2008). Being directly based on the experimental data, these methods, in general, have shown higher performance as compared to traditional methods for predicting ncRNAs and CREs. We expect a wider application of this approach in analyzing HTS data not only for the functional annotation of the genome, but also to unravel the spatiotemporal activity of these annotated elements across different cell types.

Read profiles have in particular been employed for the analysis of small RNA-seq data. However, equivalent strategies can also be employed for total RNA-seq or polyA RNA-seq that includes long ncRNAs (lncRNAs) and mRNAs. Read profiles from these transcripts can include patterns, for example originating from alternative splicing mechanisms (both coding as well as non-coding). Furthermore, with growing amount of new applications of sequencing (such as CLIP-seq and PAR-CLIP), we anticipate that the need for comparing read profiles would increase. Indeed, two recent methods (PARma and PARalyzer) utilized the patterns obtained after mapping short reads from PAR-CLIP to determine the miRNA target sites (Corcoran et al., 2011; Erhard et al., 2013). Here, PAR-CLIP is used to sequence RNA bound by cellular RNA-binding proteins (RBPs) and microRNA-containing ribonucleoprotein complexes (miRNPs) (Hafner et al., 2010). Both PARma and PARalyzer start by identifying read clusters, which exhibit T to C conversions that is an important characteristic of reads corresponding to actual binding sites (Hafner et al., 2010). Next, a computational model compares the actual rate of T to C conversions within each read cluster with that of the background. A seed region within the read cluster having conversion rate above a threshold, along with presence of motif and generality of seed across many read clusters is defined as potential miRNA binding site (Erhard et al., 2013; Corcoran et al., 2011).

### Conflict of interest statement

The authors declare that the research was conducted in the absence of any commercial or financial relationships that could be construed as a potential conflict of interest.
